# Supplementary material for: MetaRibo-Seq measures translation in microbiomes
Source: Nat Commun. 2020 Jun 29;11:3268. doi: 10.1038/s41467-020-17081-z (PMC7324362; doi:10.1038/s41467-020-17081-z)
Supplement: Supplementary file 10 — Supplementary Data 7 [file 41467_2020_17081_MOESM10_ESM.zip › File2/Confidence_High_Taxonomy/59097_out.krona.html]

Javascript must be enabled to view this page.

members
magnitude
magnitudeUnassigned
count
unassigned
taxon
rank

59097\_out

6

6
2
superkingdom

phylum
4
976

class
4
200643

171549
4
order

4
815
family

4
816

SRS013638\_contig\_number\_18864
genus
1

3
820

SRS017821\_contig\_number\_27028SRS143372\_contig\_number\_11293SRS148091\_contig\_number\_contig-100\_967.97733
species

2
1239
phylum

class
186801
2

order
186802
2

species

SRS077127\_contig\_number\_16580
1897048
1

family
1
541000

216851
1
genus

1
1946504
species

SRS043768\_contig\_number\_29600
